# The Hydroxyl at Position C1 of Genipin Is the Active Inhibitory Group that Affects Mitochondrial Uncoupling Protein 2 in Panc-1 Cells

**DOI:** 10.1371/journal.pone.0147026

**Published:** 2016-01-15

**Authors:** Yang Yang, Yifu Yang, Jianwei Hou, Yue Ding, Tong Zhang, Yong Zhang, Jianying Wang, Chenchen Shi, Wenwei Fu, Zhenzhen Cai

**Affiliations:** 1 Laboratory of Immunology and Virology, Experiment Center For Science and Technology, Shanghai University of Traditional Chinese Medicine, Shanghai, China; 2 Experiment Center for Teaching and Learning, Shanghai University of Traditional Chinese Medicine, Shanghai, China; 3 Innovation of Traditional Chinese Medicine Laboratory, College of Traditional Chinese Medicine, Shanghai University of Traditional Chinese Medicine, Shanghai, China; Louisiana State University Health Sciences Center, UNITED STATES

## Abstract

Genipin (GNP) effectively inhibits uncoupling protein 2 (UCP2), which regulates the leakage of protons across the inner mitochondrial membrane. UCP2 inhibition may induce pancreatic adenocarcinoma cell death by increasing reactive oxygen species (ROS) levels. In this study, the hydroxyls at positions C10 (10-OH) and C1 (1-OH) of GNP were hypothesized to be the active groups that cause these inhibitory effects. Four GNP derivatives in which the hydroxyl at position C10 or C1 was replaced with other chemical groups were synthesized and isolated. Differences in the inhibitory effects of GNP and its four derivatives on pancreatic carcinoma cell (Panc-1) proliferation were assessed. The effects of GNP and its derivatives on apoptosis, UCP2 inhibition and ROS production were also studied to explore the relationship between GNP’s activity and its structure. The derivatives with 1-OH substitutions, geniposide (1-GNP1) and 1-ethyl-genipin (1-GNP2) lacked cytotoxic effects, while the other derivatives that retained 1-OH, 10-piv-genipin (10-GNP1) and 10-acetic acid-genipin (10-GNP2) exerted biological effects similar to those of GNP, even in the absence of 10-OH. Thus, 1-OH is the key functional group in the structure of GNP that is responsible for GNP’s apoptotic effects. These cytotoxic effects involve the induction of Panc-1 cell apoptosis through UCP2 inhibition and subsequent ROS production.

## Introduction

Genipin (GNP) is derived from the dry fruits of *Gardenia jasminoides* Ellis, which has long been used in traditional Chinese medicine due to its positive effects on inflammation and hepatic disorders [1]. GNP has been reported to have anti-inflammatory [2, 3], anti-angiogenic, anti-thrombotic [4], anti-diabetic [5, 6], choleretic [7], liver protective [8], and neurotrophic activities [9]. In particular, it has been shown to promote apoptosis in rat glioma C6 cells [10], human prostate cancer cells(PC3) [11], human cervical cancer cells (HeLa) [12], human hepatocarcinoma Hep3B cells and rat hepatoma FaO cells [13], human non-small-cell lung cancer cells (H1299) [14], human leukemia K562 cells [15], and human pancreatic adenocarcinoma PaCa44, PaCa3 and Panc-1 cells [16]. Furthermore, GNP inhibits drug resistance in cancer cells by increasing the susceptibility to oxidative stress and cytotoxic agents, and all of these effects are related to its high affinity for uncoupling protein-2 (UCP2) [17–19].

The uncoupling proteins (UCPs) are mitochondrial anion transporter proteins that are localized to the inner mitochondrial membrane [20]. Several studies have demonstrated that UCP2 is over-expressed in cancer cells, which attenuates oxidative stress by increasing proton influx into the mitochondrial matrix and by decreasing mitochondrial superoxide generation and electron leakage, supporting the notion that the mitigation of oxidative stress is an adaptive mechanism established by cancer cells for the homeostatic maintenance of reactive oxygen species (ROS) [21, 22]. The inhibition of UCP2 via GNP increases the generation of mitochondrial superoxide ions, particularly in cancer cells, leading to apoptosis, cell cycle arrest, autophagy, apoptosis and the prevention of chemoresistance.

However, the mechanism by which GNP inhibits UCP2 remains unknown, and the relationship between its chemical structure and biological effects has not yet been determined. GNP is obtained from geniposide (1-GNP1) as the product of the hydrolysis of glucose at the C1 site by bacterial enzymes termed *β*-D-glycosidases, which exist in the intestines and liver. An *in vitro* study has found that 1-GNP1 does not induce apoptosis in hepatoma cells, in contrast with GNP [8]. In addition, in the presence of an equimolar amount of glycine, GNP can dimerize to generate genipocyanin G1, which is a blue-pigmented, highly conjugated dimeric adduct with the ability to cross-link proteins [23–25]. Cytochrome c has also been shown to be cross-linked by GNP, forming oligomers in a process that likely involves the generation of complexes via the reaction of two primary amine groups from separate proteins with the two hydroxyls of GNP [6]. GNP derivatives that lack the hydroxyl at the C1 position (1-OH) or at the C10 position (10-OH) may have decreased cross-linking abilities because 1-OH and 10-OH are likely necessary for the generation of oligomers [26]. In the present study, GNP and four GNP derivatives with 1-OH or 10-OH substitutions were prepared. Differences in the inhibitory effects of GNP and its four derivatives on pancreatic carcinoma cell (Panc-1) proliferation were assessed to explore the relationship between GNP’s activity and structure. For further mechanistic analysis, the effects of GNP and its derivatives on apoptosis, UCP2 inhibition and ROS production were also examined. Based on the evidence accumulated to date, 1-OH is the key site for GNP’s biological effects, whereas the apoptosis that is induced by GNP and its derivatives is related to UCP2 inhibition in Panc-1 cells and the induction of ROS production.

## Materials and Methods

### Preparation of GNP and its derivatives

**Fig 1:**
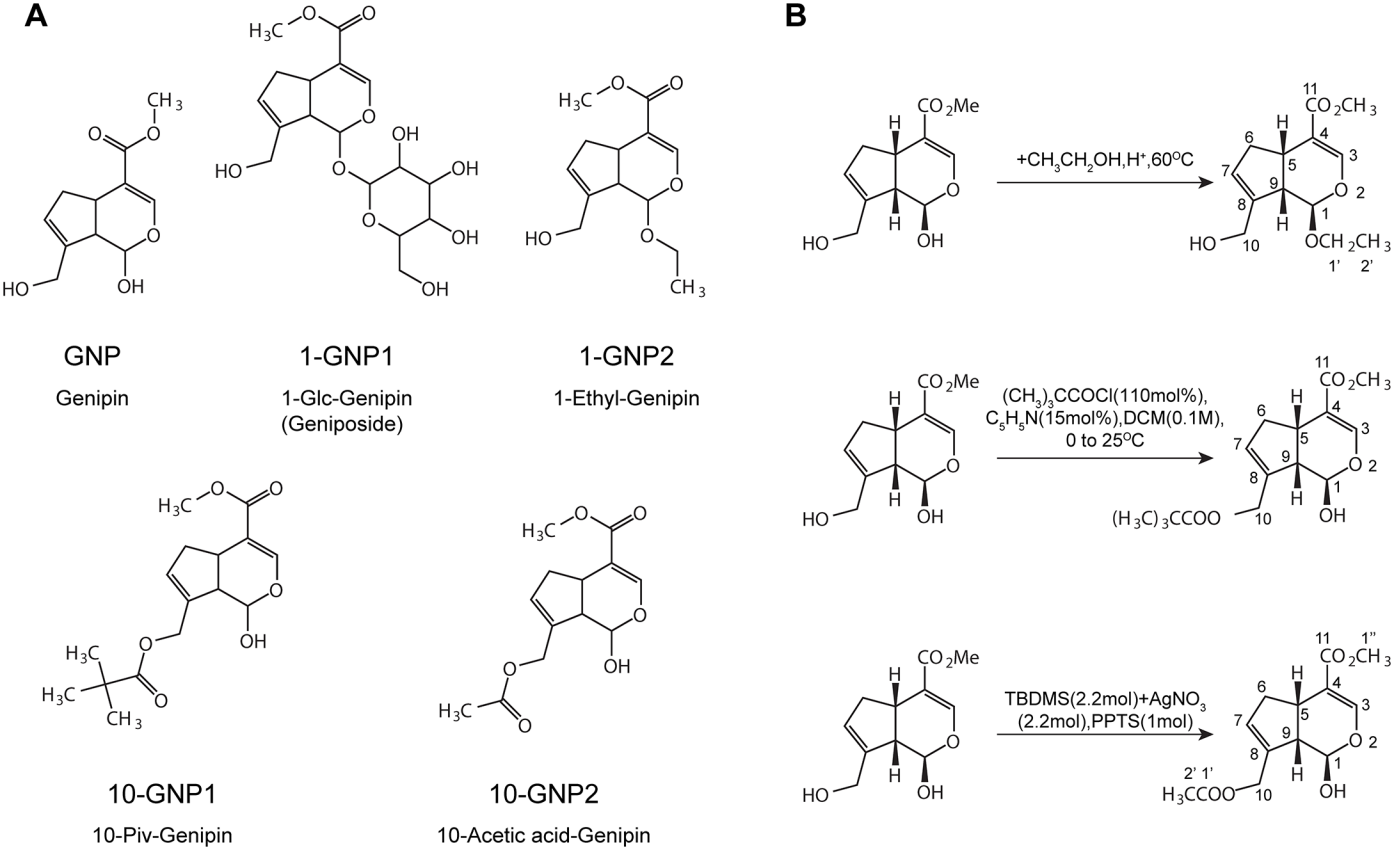
Chemical structures of the GNP derivatives and the reactions used to synthesize them. Chemical structures of GNP and its derivatives. (B) The synthetic processes of 1-GNP2, 10-GNP1 and 10-GNP2.

Four GNP derivatives were isolated and synthesized to identify the active groups in the structure of GNP. Based on their structural characteristics, GNP and the four derivatives, 1-GNP1, 1-ethyl-genipin (1-GNP2), 10-piv-genipin (10-GNP1) and 10-acetic acid-genipin (10-GNP2) were divided into two groups. The first group included GNP, 10-GNP1 and 10-GNP2; these compounds contained 1-OH, but 10-OH was replaced by another group. The second group included 1-GNP1 and 1-GNP2, in which 1-OH was replaced by another group. The structures of GNP and its derivatives are shown in Fig 1A.

1-GNP1 was isolated from *Gardenia jasminoides* Ellis as described in our previous study [27, 28]. GNP was prepared by the hydrolysis of 1-GNP1 using a previously reported procedure [29]. **1-**GNP2, 10-GNP1, and 10-GNP2 were synthesized in our laboratory. Their purity was analyzed by high-performance liquid chromatography (HPLC). The infrared (IR), ESI-MS, UV, ^1^H-NMR and ^13^C-NMR spectra were collected to identify their structures.

**1-GNP2** Five grams of GNP was dissolved in 100 ml ethanol with one drop of concentrated hydrochloric acid. The mixture was blended at 60°C for 6 h, brought to pH 7 with 1 M sodium hydroxide and dried under vacuum. Extraction was performed using 100 ml of ethyl acetate, and the ethyl acetate phase was collected, then washed with 100 ml of saline water and dried. Further purification was completed using a silica gel column (15 mm*100 mm, 70 g) with an eluent (chloroform:ethyl acetate = 3:1, v/v), which was detected by thin-layer chromatography (TLC), collected, and dried. The reaction equation is shown in Fig 1B. We obtained 0.3180 g of white powder.

**10-GNP1** We dissolved 0.5 g GNP in 22 ml dichloromethane with 0.267 ml pyridine. Then, 0.299 ml trimethylacetyl chloride was added under an N_2_ atmosphere at 0°C, and the mixed solution was stirred for 12 h. The mixture was then dried with a rotary evaporator and dissolved in 20 ml of ethyl ether. The solution was washed with 20 ml of saturated ammonium chloride solution, saturated sodium chloride solution and 5% CuSO_4_ solution, sequentially. The ethyl ether layer was collected and dried. A silica gel column (15 mm*100 mm, 70 g) and an eluent (ethyl acetate:petroleum ether = 1:4, v/v) were used for purification of the product. The eluent was detected by TLC, collected and dried. The reaction equation is shown in Fig 1B. We obtained 0.2530 g of white powder.

**10-GNP2** We dissolved 0.68 g GNP in 25 ml of dimethylformamide with 9.9 g TBS t-butyldimethylsilyl (TBDMS) and 11.2 g AgNO_3_. The solution was placed into a 50-ml round flask and stirred overnight at room temperature. After the solution was filtered, it was washed with water. The solution was then dried, dissolved in 25 ml of ethanol, and stirred for 6 h at room temperature with 16.6 g (0.066 mol) pyridinium p-toluenesulfonate (PPTS). Next, it was washed with water, dried, dissolved in 10 ml of dichloromethane and blended for 3.5 h at room temperature with 0.306 g (0.03 mol) acetic anhydride, 0.109 g (0.05 mol) 4-dimethylaminopyridine (DMAP) and 0.1 ml triethylamine. The product was then washed with water and dried using a rotary evaporator. The dried product was stirred for another 3.5 h in nBu_4_NF and acetic acid solvent, washed with water, and dried again using a rotary evaporator. Purification was achieved using a silica gel column (15 mm*100 mm, 70 g) with an eluent (petroleum ether:ethyl acetate = 4:1, v/v), which was detected by TLC, collected, and dried. The reaction equation is shown in Fig 1B. We obtained 0.2187 g of white powder.

### Cell culture

The human pancreatic adenocarcinoma Panc-1 cell line and the hepatocellular carcinoma HepG2 cell line were obtained from the Cell Bank of Type Culture Collection of the Chinese Academy of Sciences, Shanghai Institute of Cell Biology (Shanghai, China) and have been described previously [30–33]. The Panc-1 cells were cultured in RPMI 1640 supplemented with 1% antibiotic-antimycotic solution (100×, Gibco), 10% heat-inactivated fetal bovine serum, and 0.5 μM 2-mercaptoethanol and incubated at 37°C under 5% CO_2_. The HepG2 cells were cultured in high-glucose DMEM with stable L-glutamine supplemented with 1% antibiotic-antimycotic solution (100×, Gibco), 10% heat-inactivated fetal bovine serum, and 0.5 μM 2-mercaptoethanol and incubated at 37°C under 5% CO_2_. The cells were harvested two times per week with trypsin/ethylenediamine tetraacetic acid for passaging and were seeded in 100-mm dishes. Cells were seeded at a density of 5×10^3^ cells/well in 96-well plates to assess cell proliferation and at 1.2×10^5^ cells/well in 6-well plates to examine apoptosis, cell morphology, ROS levels, the mitochondrial membrane potential and cell cycle arrest.

### Cell viability assay

The crystal violet assay was performed to assess cell viability. Panc-1 cells and HepG2 cells were seeded at densities of 5×10^3^ and 3×10^4^ cells/well, respectively, in 96-well plates and incubated for 24 h. They were then exposed to GNP or its derivatives (at 50, 100, 200, and 400 μM) for 72 h. Next, the cells were fixed and stained with 0.03% crystal violet (w/v) in 20% methanol. The dye was solubilized in lysis buffer (0.8979 g sodium citrate and 1.25 ml of 1N HCl in 98.05 ml of 47.5% ethanol), and the absorbance was spectrophotometrically measured at 540/630 nm to assess cell growth. All data are provided as the mean of three independent experiments.

### Morphological observation of cells and Hoechst staining

Panc-1 cells were seeded in 6-well plates (1.2×10^5^ cells/well) for 24 h, treated with GNP or its derivatives (at 200 μM) for 72 h at 37°C, and then directly observed by inverted phase-contrast microscopy. Another group of cells was treated with GNP or its derivatives (200 μM) for 48 h. They were then washed by the addition of PBS, fixed in 4% paraformaldehyde at room temperature for 30 min and resuspended in PBS before the placement of cover slips. Adhered cells were incubated with Hoechst 33258 for 20 min at 37°C. The cover slips were rinsed with PBS and imaged by fluorescence microscopy.

### Flow cytometric analysis of cell apoptosis

Panc-1 cells were seeded in 6-well plates (1.2×10^5^ cells/well). After 24 h, they were treated with GNP or its derivatives (at 200 μM) for 48 h at the indicated concentrations. Next, the cells were fixed with 2% paraformaldehyde in PBS for 30 min at room temperature. The cells were then washed twice with PBS and stained with annexin V/FITC in binding buffer at room temperature for 10 min in the dark. Washed cells that were suspended in binding buffer were stained with propidium iodide (PI) in binding buffer. Finally, cell fluorescence was assessed by flow cytometry (FACSCalibur; BD Biosciences). Data analysis was performed using CellQuest software.

### Flow cytometric analysis of cell cycle progression

To further examine the effects of these compounds on the induction of cell cycle activity, Panc-1 cells were incubated for 24 h in 6-well plates with GNP and its derivatives (at 200 μM). The cells were then harvested, washed by the addition of PBS, and fixed by incubation in ice-cold 70% ethanol overnight at 4°C. Suspended cells were centrifuged, the supernatant was discarded, and the cells were then resuspended in PI staining solution (100 μg/ml RNase A and 40 μg/ml PI) for 30 min at 37°C. The samples were assessed by flow cytometry (FACSCalibur; BD Biosciences). Data analysis was performed using Modfit software.

### UCP2 gene over-expression and silencing

UCP2 over-expression experiments were conducted by using a pCMV expression vector containing human UCP2 cDNA and Lipofectamine 2000 transfection reagent (Life Technologies; Invitrogen). Successfully transfected cells were selected with G418 (Geneticin). Cells transfected with an empty pCMV vector were employed as negative controls (mock). The transfection efficiency was greater than 30%, as previously determined by cytofluorimetric analysis using a pGFP vector (Figure A in S1 File). UCP2 over-expression was measured by Western blotting (Figure B in S1 File) [34]. Cells were incubated for 72 h with three levels (40, 120, and 200 μM) of GNP and its derivatives to determine their effects on UCP2 over-expression and cell viability.

UCP2 silencing experiments were performed with a specific small interfering (si) (5′-CACCTTTCCTCTGGATACTGCTAAA-3′) RNA that targeted UCP2 mRNA and a non-targeting (NT) siRNA (5′-CAGUCGCGUUUGCGACUGG-3′); both siRNAs were acquired from Life Technologies (Invitrogen). Transfection of cells was performed for 24 h using siRNAs at final concentrations of 50, 100, and 150 nM with Lipofectamine RNAiMAX reagent. The transfection efficiency was previously assessed by qPCR (Real-time Quantitative PCR Detecting System) (Figure C in S1 File) and the down-regulation of UCP2 expression was measured by Western blotting (Figure B in S1 File) [35]. Panc-1 cells were incubated for 72 h with three levels (40, 120, 200 μM) of GNP and its derivatives to determine their effects on UCP2 silencing and cell viability.

### Analysis of intracellular reactive oxygen species

Intracellular ROS generation was assessed by using a 2’,7’-dichlorofluorescein diacetate (DCF-DA) probe. Cells were seeded in 96-well plates (5×10^3^ cells/well) or 6-well plates (1.2×10^5^ cells/well) and treated with GNP or its derivatives (at 200 μM) after 24 h. Then, the cells were incubated with 10 μM DCF-DA was using for cells incubation for 15 min at 37°C. After staining, the cells were examined by fluorescence microscopy, flow cytometry and a multi-color fluorescence scanning imaging system. All data are shown as the mean of three independent experiments.

### Determination of mitochondrial membrane potential

A lipophilic cation probe, 5,5',6,6'-tetrachloro-1,1',3,3'-tetraethylbenzimidazol- carbocyanine iodide (JC-1), was employed to assess the mitochondrial membrane potential. This probe is capable of specifically entering into mitochondria, so it is more effective than rhodamine and other carbocyanines, and it exhibits a reversible fluorescence color change from green (FL1) to orange (FL2) with increasing membrane potential. Panc-1 cells were digested in sterile centrifuge tubes using a trypsin-EDTA solution (Gibco), and then washed twice with PBS and resuspended in 0.5 ml KRBH buffer containing 1% (w/v) BSA. After pre-incubation for 20 min with GNP or its derivatives (at 200 μM), they were incubated with JC-1 for 20 min at 37°C. The cells were then washed two times and resuspended in PBS. Flow cytometry (FACSCalibur; BD Biosciences) was performed immediately to measure cell fluorescence. Data analysis was conducted using CellQuest software.

### Statistical analysis

The data are shown as the mean ± standard error (SE). Student’s t-test was used to conduct statistical analyses, and Prism 5 software was used to generate graphs. Statistical significance was set at a p<0.05.

## Results

### Identification of GNP and its derivatives

1-GNP1 is a white powder, and its purity was determined to be as high as 98%, as determined by HPLC analysis. Its structure was determined by comparing HPLC and ESI-MS spectra with the reference standard (1-GNP1, CAS Number: 24512-63-8, Tauto Biotech Industries Ltd., China). In positive-mode UPLC-MS/MS spectra, a major ion weight [M+Na]^+^ of 411.1 with product ions at m/z 379.1, 217.0 and 349.0 was detected, whereas in negative mode, a deprotonated weight [M-H]^-^ of 387.0 with product ions at m/z 225.1 and 123.0 was detected. The derived formula of 1-GNP1 was C_17_H_24_O_10_, and its structure is shown in Fig 1A.

GNP is a white powder and its purity was determined to be as high as 98%, as determined by HPLC analysis. Its structure was determined by comparing HPLC and ESI-MS spectra with the reference standard (GNP, CAS Number: 6902-77-8, Wako Pure Chemical Industries Ltd, Japan). In negative-mode UPLC-MS/MS spectra, a deprotonated weight [M-H]^-^ of 225.1 and product ions at m/z 207.1, 147.0, 123.0 and 101.0 were detected. The derived formula of GNP was C_11_H_14_O_5_, and its structure is shown in Fig 1A.

1-GNP2 is a white powder. The synthesis reaction equation is shown in Fig 1B. Its purity was determined to be as high as 98%, as calculated using the area normalization method of HPLC. The ESI-MS, ^1^H-NMR and ^13^C-NMR spectra were collected for structural identification. A pseudo molecular ion weight [M+H]^+^ of 255.3 with product ions at m/z 209.3, 177.2, and 149.3 was detected in positive-mode UPLC-MS/MS spectra. The derived formula of 1-GNP2 was C_13_H_18_O_5_, and its structure is shown in Fig 1A. The ^1^H-NMR and ^13^C-NMR spectra and data are shown in Figure D and Figure E in S1 File.

10-GNP1 is a white powder. The synthesis reaction equation is shown in Fig 1B. The yield was 71.3%, and the purity was up to 98%, as calculated using the area normalization method of HPLC. The ESI-MS, ^1^H-NMR and ^13^C-NMR spectra were collected to determine its structure. A deprotonated weight [M-H]^-^ of 309.1 with product ions at m/z 207.1, 175.1, 147.1 and 101.1 was detected in negative-mode UPLC-MS/MS spectra. The derived formula of 10-GNP1 was C_16_H_22_O_6_, and its structure is shown in Fig 1A. The ^1^H-NMR and ^13^C-NMR spectra and data are shown in Figure F and Figure G in S1 File. 10-GNP2 is a white powder. The synthesis reaction equation is shown in Fig 1B. The yield was 70.0%, and the purity was up to 99%, as calculated using the area normalization method of HPLC-VWD. The ESI-MS, ^1^H-NMR and ^13^C-NMR spectra were collected to determine its structure. A deprotonated weight [M-H]^-^ of 267.2 with product ions at m/z 207.1, 175.1, 147.2, and 101.2 was observed. The derived formula of 10-GNP2 was C_13_H_16_O_6_, and its structure is shown in Fig 1A. The ^1^H-NMR and ^13^C-NMR spectra and data are shown in Figure H and Figure I in S1 File.

### Inhibitory effects of GNP and its derivatives on Panc-1 cell growth

**Fig 2:**
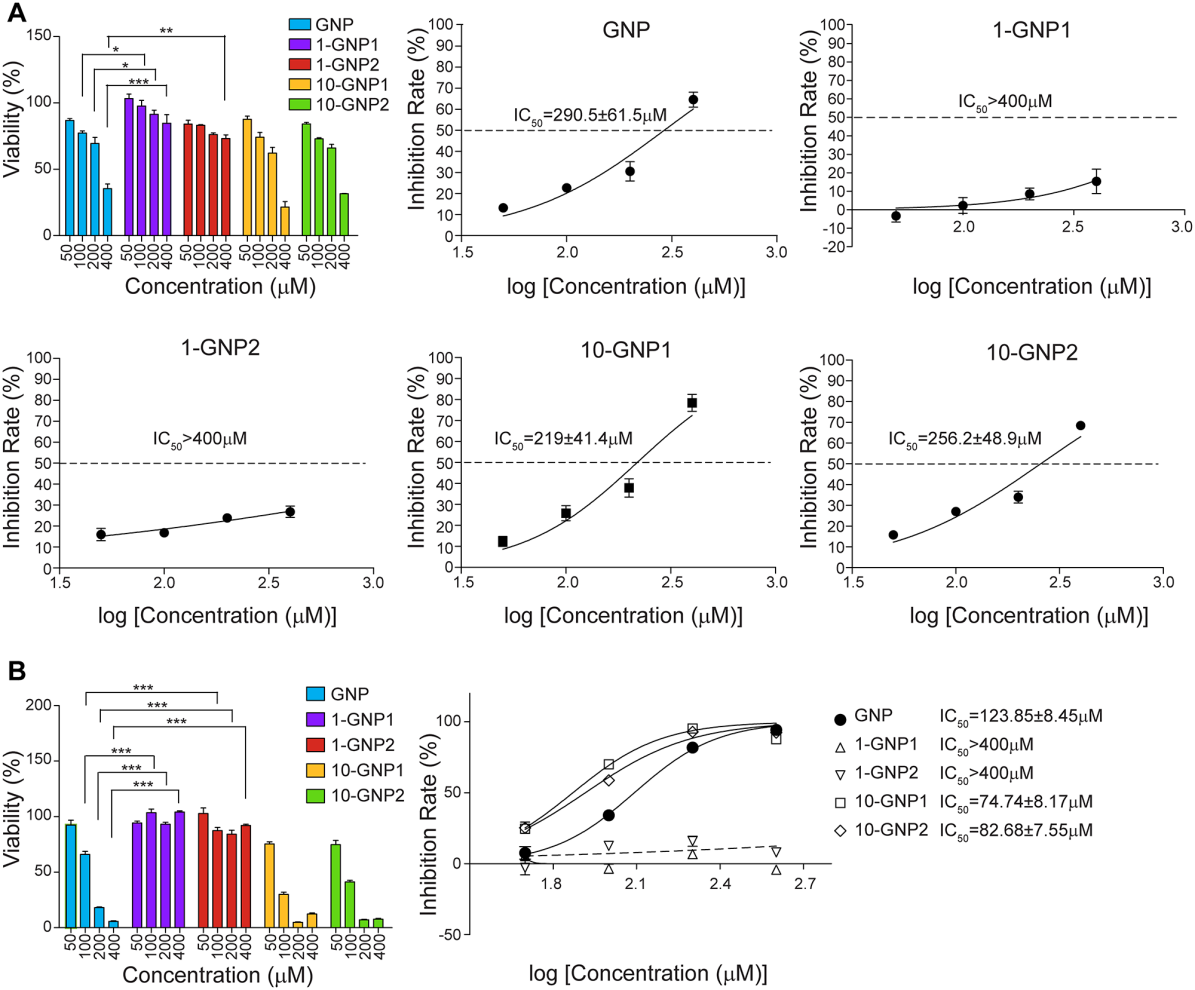
Cytotoxicity of GNP and its derivatives on Panc-1 cells and HepG2 cells. The effects of GNP and its derivatives on the Panc-1 cell (A) and HepG2 cell(B) survival rate at concentrations of 50, 100, 200 and 400 μM after 72 h, as determined by crystal violet colorimetric assay. The values are the means (±SE) of three independent experiments. Statistical analysis was performed by comparing the viability of control cells with that of cells treated with GNP (*p<0.05, **p<0.01 and ***p<0.001).

GNP has been reported to exert a wide range of inhibitory effects on tumor growth and also to promote apoptosis. The Panc-1 cell line was used to compare the effects of GNP and its derivatives (1-GNP1, 1-GNP2, 10-GNP1 and 10-GNP2) on tumor cell survival rate. Because GNP's pharmacological mechanism is associated with the mitochondrial transmembrane protein UCP2, the crystal violet method was used to detect the effects of GNP and its derivatives on the Panc-1 cell survival rate to prevent a nonspecific reaction from occurring between GNP or its derivatives and succinate dehydrogenase, which is used in the MTT and CCK8 (Cell Counting Kit-8) assays. After Panc-1 cells were incubated with GNP and its derivatives (50, 100, 200, and 400 μM) for 72 h, the derivatives were divided into two structure-based groups that exhibited different effects on Panc-1 cell growth. 10-GNP1 and 10-GNP2, which had 10-OH substitutions but retained 1-OH, had the same effects as GNP in significantly reducing Panc-1 cell viability. GNP, 10-GNP1 and 10-GNP2 showed cytotoxic and antiproliferative effects on Panc-1 cells in a dose-dependent manner. In contrast, 1-GNP1 and 1-GNP2, which had 1-OH substitutions, had no effects on cell viability. The concentrations of GNP and its derivatives needed to inhibit 50% of the cells (IC_50_) are also shown in Fig 2A. The IC_50_ values of GNP and the 10-OH-substituted derivatives ranged from 219 μM to 290.5 μM, but the values of the 1-OH-substituted derivatives exceeded 400 μM. Further, to demonstrate that the inhibition of viability was not cell line dependent, HepG2 cells were used to compare the inhibitory effects of GNP and its derivatives, and the results were consistent with those for the Panc-1 cells (Fig 2B). The IC_50_ values of GNP and the 10-OH-substituted derivatives for the HepG2 cell ranged from 74.7 μM to 123.9 μM, whereas the values of the 1-OH-substituted derivatives exceeded 400 μM.

### Cellular apoptosis induced by GNP and its derivatives

**Fig 3:**
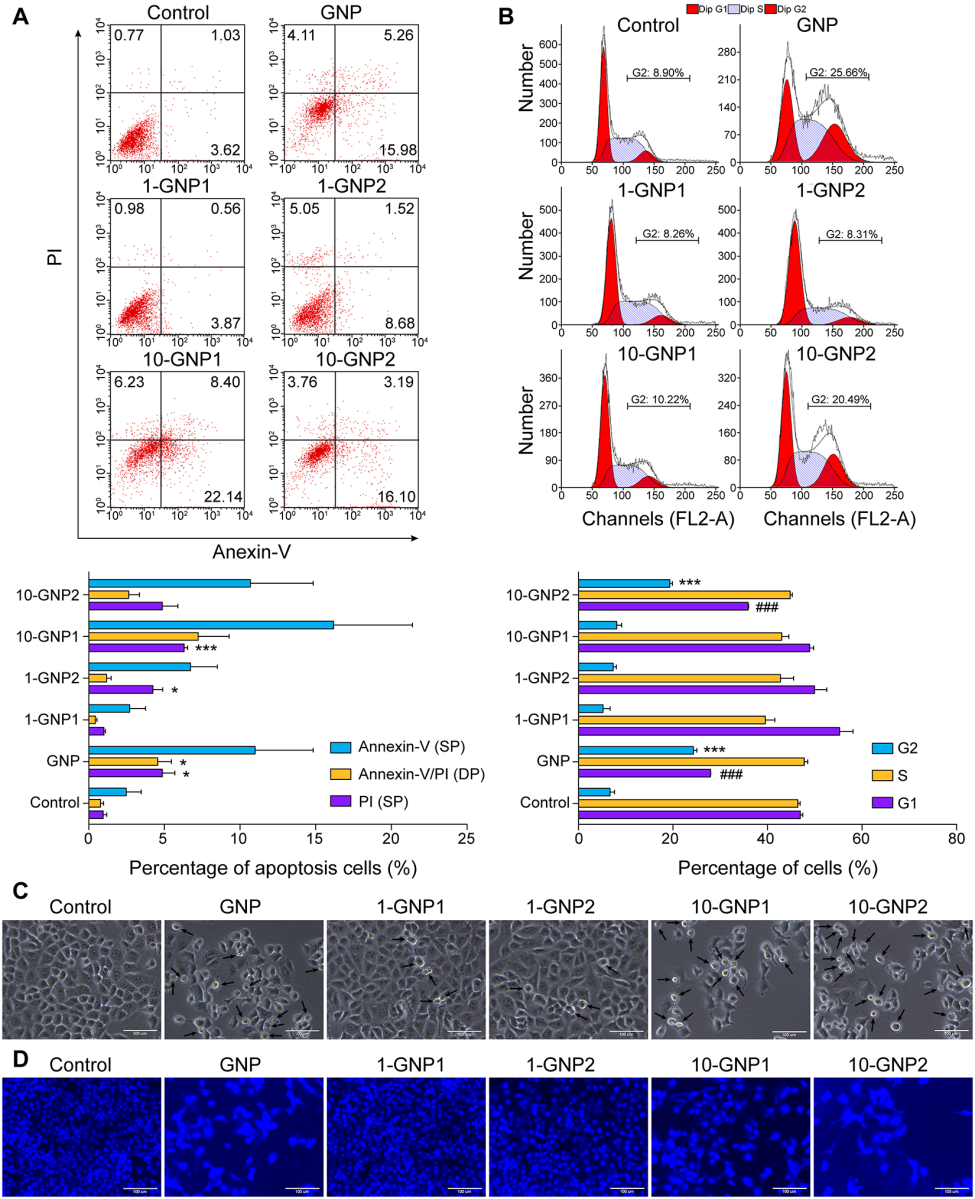
Effects of GNP and its derivatives on apoptosis and cell cycle arrest in Panc-1 cells. Cells apoptosis induced by GNP and its derivatives was analyzed using the annexin V (FITC)/PI binding assay by flow cytometry. The values are the means (±SE) of three independent experiments. Statistical analysis was performed by comparing the annexin V and PI indexes in cells that were treated with GNP and its derivatives with those in cells in the control group (*p<0.05, **p<0.01, and ***p<0.001). (B) Panc-1 cells morphology was analyzed using a microscope (magnification, 400×) after the cells were treated with GNP and its derivatives. The cells treated with GNP, 10-GNP1 and 10-GNP2 exhibited several characteristic morphological changes, including the appearance of more rounded, shrunken, and suspended cells. In contrast, the morphologies of those treated with 1-GNP1 and 1-GNP2 were normal, with round and homogeneous nuclei similar to those observed in Panc-1 cells that were cultured in control medium. (C) The effects of GNP and its derivatives on Panc-1 cell cycle progression were determined by flow cytometric analysis. The values are the mean (±SE) of three independent experiments. Following treatment with GNP or 10-GNP2, the percentage of cells at G_2_/M was significantly increased compared with control cells; this effect was accompanied by a significant decrease in the percentage of G_1_ cells (*** p<0.001). (D) Morphological changes in cell nuclei and chromosomes were observed by Hoechst 33258 staining and fluorescence microscopy (magnification, 200×).

To assess the effects of GNP and its derivatives on the induction of apoptosis, Panc-1 cells were exposed to GNP and its derivatives at 200 μM for 48 h. Apoptosis (annexin V/PI) analysis was performed using flow cytometry. The apoptosis rate of the Panc-1 cells was analyzed using CellQuest software. The apoptosis rates of the 10-GNP1- and 10-GNP2-treated cells were similar to that of the GNP-treated cells. Furthermore, FACS analysis revealed the absence of Panc-1 apoptosis after 1-OH substitution in 1-GNP1 and 1-GNP2, and no differences were observed relative to control cells (Fig 3A).

### Effects of GNP and its derivatives on cell cycle progression

A comparison of the percentages of control cells and cells treated with GNP and its derivatives for 24 h in the G_0_/G_1_, S, and G_2_/M phases was performed to analyze cell cycle distribution. In the control group, apoptotic (sub G_0_/G_1_) cells were not assessed, and a total of 6.67±1.97% of all viable control cells was found to be in G_2_/M phase. After 24 h of treatment with 200 μM GNP, the G_2_/M population increased to 24.3±1.49% (a nearly 4-fold increase) of the total number of viable cells. This change was accompanied by decreases in the G_1_ and S populations. Treatment with GNP significantly altered the cell cycle distribution and increased the number of apoptotic cells, demonstrating that the decrease of cell viability was caused by the induction of apoptosis and was related to cell cycle arrest. The percentages of cells at G_2_/M were compared among the GNP derivatives and were as follows: 5.19±3.04% (1-GNP1), 7.35±1.29% (1-GNP2), 8.11±2.06% (10-GNP1), and 19.32±1.1% (10-GNP2) (Fig 3C). These results verify the conclusions that apoptosis induced cell death and that GNP and 10-GNP2 caused G_2_/M arrest in the Panc-1 cells. GNP has also been reported to induce the accumulation of K562 cells at the G_2_/M phase [14]. We obtained the same results for Panc-1 cells, and the effects of the derivatives on cell cycle arrest were consistent with the induction of apoptosis in the Panc-1 cells. However, the proportion of 10-GNP1-treated cells arrested at the G2/M phase at 24 h was 8.11±2.06%, which was not significantly different from the proportions of 1-GNP1- and 1-GNP2-treated cells that were accumulated at G2/M. These results may be due to the different chemical structures of 10-GNP1, 10-GNP2 and GNP. Because 10-OH was replaced with trimethylacetyl, which is a large group, the solubility of 10-GNP1 in the cell culture medium was much lower than that of 10-GNP2 and GNP. In addition, the different chemical structures would cause 10-GNP1, 10-GNP2 and GNP to have different absorption rates for Panc-1 cells. The G2/M-arrested population of 10-GNP1-treated cells was not as large as those of the 10-GNP2- and GNP-treated cells at 24 h, but the population size increased to 34.06% at 48 h. These results suggest that different amounts of time were required for 10-GNP1, 10-GNP2 and GNP to reach their full effects. In this manuscript, we present the sizes of the G_2_/M-arrested populations of 10-GNP1-, 10-GNP2- and GNP-treated Panc-1 cells at the same time point of 24 h.

Morphological changes were evaluated by microscope after Panc-1 cells were treated with GNP or its derivatives at 200 μM for 72 h. The number of cells was significantly reduced after treatment with GNP, 10-GNP1 and 10-GNP2, and more rounded, shrunken, and suspended cells with typical apoptotic morphological changes were observed (Fig 3C arrows) in these treated cells compared to in untreated control cells. However, the morphology of the cells that were treated with 1-GNP1 and 1-GNP2 was similar to that of Panc-1 cells cultured in control medium; these cells exhibited the normal characteristic of round and homogeneous nuclei (Fig 3C). To better clarify the changes in cell morphology induced by GNP and its derivatives during apoptosis, Hoechst 33258 staining was used to assess nuclear morphology in Panc-1 cells that were treated for 48 h with GNP or its derivatives at 200 μM. Increased nuclear size was observed in the GNP-, 10-GNP1- and 10-GNP2-treated cells; this effect could be related to G_2_/M arrest. In contrast, 1-GNP1- and 1-GNP2-treated cells had morphologically normal nuclei, similar to those in the controls (Fig 3D).

### UCP2 partially mediated the inhibitory effects of GNP and its derivatives on Panc-1 cells

**Fig 4:**
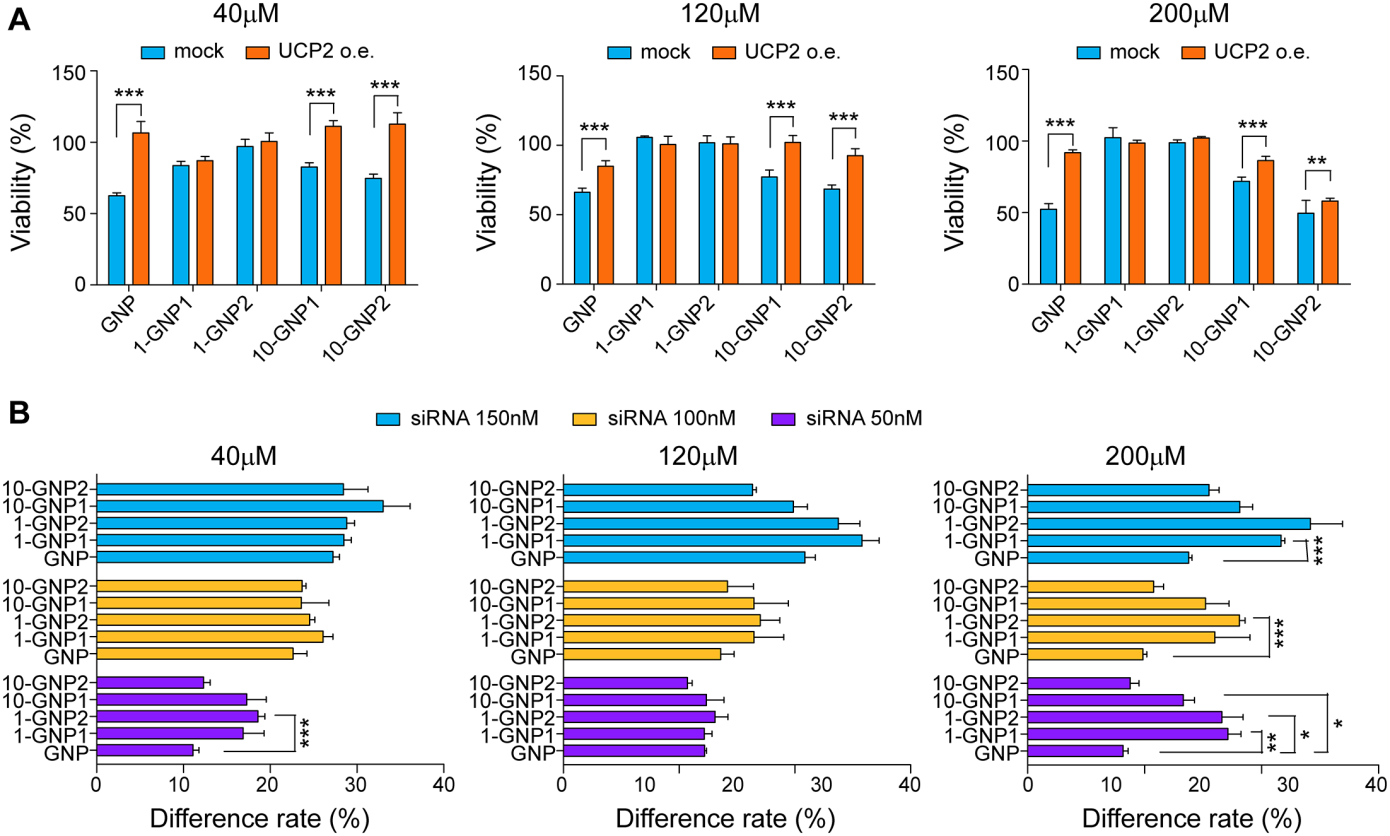
UCP2 partially mediated the inhibitory effects of GNP and its derivatives in Panc-1 cells. The inhibitory effects of GNP, 10-GNP1 and 10-GNP2 were significantly blocked by UCP2 over-expression in Panc-1 cells (***p<0.001 and **p<0.01), whereas 1-GNP1 and 1-GNP2 exhibited no inhibitory effects on Panc-1 cells with either normal expression or over-expression of UCP2. (B) Differences in the inhibitory effects of GNP and its derivatives on UCP2-silenced Panc-1 cells and normal Panc-1 cells were determined using crystal violet colorimetric assay. The rate differences (Y values) of GNP, 10-GNP1 and 10-GNP2 were significantly smaller than those of 1-GNP1 and 1-GNP2 (***p<0.001, **p<0.01, and *p<0.05). For all experiments, the values are the mean (±SE) of three independent experiments performed in triplicate.

GNP is an effective inhibitor of UCP2. UCP2 provides protection against oxidative stress by increasing the flow of protons across the mitochondrial matrix, thereby increasing the efficiency of electron flow through the respiratory complexes. GNP can induce apoptosis in many types of cancer cells by inhibiting UCP2 function. To determine whether the effects of GNP and its derivatives on UCP2 were similar, a UCP2 over-expressing Panc-1 cell line was constructed using a pCMV expression vector. A UCP2-silenced Panc-1 cell line was also constructed using UCP2 siRNA. The different inhibitory effects induced by GNP and its derivatives on UCP2 over-expressing and low-expressing Panc-1 cells were then separately compared. The inhibitory effects of GNP, 10-GNP1 and 10-GNP2 were blocked by UCP2 over-expression in Panc-1 cells, whereas 1-GNP1 and 1-GNP2 exhibited no inhibitory effects on Panc-1 or control (mock) cells with either normal expression or over-expression of UCP2 (Fig 4A). UCP2 silencing in Panc-1 cells as mediated by siRNA resulted in the same inhibitory effect as that caused by GNP. GNP and UCP2 siRNA were assumed to affect the same site of UCP2, which may result in a significant decrease due to the superposition effect. Thus, the relationships between the GNP derivatives and UCP2 silencing were determined according to the following equation: Y = (*x*/UCP2—*x*/NT)×100%, where Y is the rate difference, *x* is the OD value of cells treated with GNP or its derivatives in UCP2 siRNA transfected cells, *UCP2* is the OD value of cells treated with UCP2 siRNA only, and *NT* is the OD value of cells treated with non-target siRNA only. The results showed that the rate differences (Y values) of GNP, 10-GNP1 and 10-GNP2 were much smaller than those of 1-GNP1 and 1-GNP2 (Fig 4B). The results of the UCP2 over-expression and silencing experiments indicate that 10-GNP1 and 10-GNP2 inhibit UCP2 function and suppress cell growth in a manner similar to GNP but that 1-GNP1 and 1-GNP2 have no such effects.

### Regulation of ROS production by GNP and its derivatives

**Fig 5:**
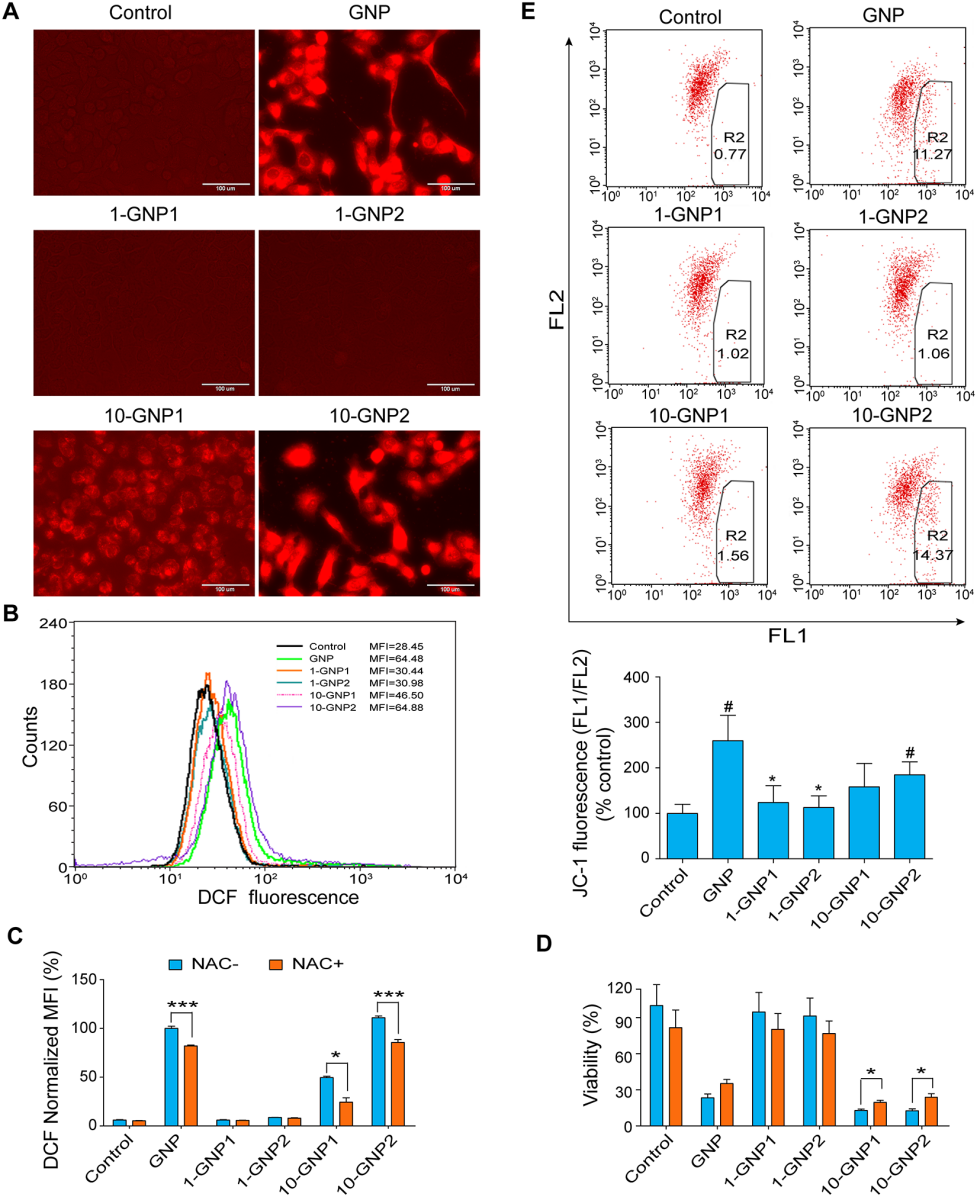
Effects of GNP and its derivatives on ROS production in Panc-1 cells. ROS production in Panc-1 cells was assessed by DCFH-DA staining. DCF fluorescence was observed under a fluorescence microscope (magnification, 400×). (B) DCF fluorescence intensity was measured by flow cytometry. (C) Panc-1 cells were pre-treated with 10 mM NAC and then treated with GNP and its derivatives at 200 μM for 24 h. DCF fluorescence intensity was measured using a multimode plate reader. Concurrent treatment with NAC significantly decreased the fluorescence intensities of the DCFH-DA reactions induced by GNP (***p<0.001), 10-GNP1 (*p<0.05) and 10-GNP2 (***p<0.001). (D) Cell viability was determined using crystal violet colorimetric assay. NAC treatment significantly increased the Panc-1 cell growth rate in the presence of 10-GNP1 and 10-GNP2 (*p<0.05). (E) The mitochondrial membrane potential of Panc-1 cells was measured using JC-1 dye. The data are expressed as the 600/535 nm fluorescence ratio. Statistical analysis: (*p<0.05), GNP derivatives *vs*. GNP; (^#^p<0.05), GNP and its derivatives *vs*. control. For all experiments, the values are the mean (±SE) of three independent experiments performed in triplicate.

ROS production in Panc-1 cells was assessed by DCFH-DA staining. DCF fluorescence intensity was significantly increased in cells that were treated with GNP, 10-GNP1, and 10-GNP2 compared with that in those treated with 1-GNP1, 1-GNP2, and control medium, as determined by fluorescence microscopy (Fig 5A) and flow cytometry (Fig 5B). ROS are important mediators of the stress response in many different types of cells. To demonstrate whether ROS participated in the induced effect, the antioxidant N-acetyl-L-cysteine (NAC) was added to Panc-1 cells that were cultured in the presence of the experimental compounds. Concurrent treatment with NAC resulted in a marked decrease in the fluorescence intensity of the DCFH-DA reactions induced by GNP, 10-GNP1, and 10-GNP2 (Fig 5C). NAC treatment significantly increased the Panc-1 cell growth rate in the presence of 10-GNP1 and 10-GNP2 (Fig 5D). However, 1-GNP1 and 1-GNP2 did not induce ROS production or the associated reactions. Additionally, an *in vitro* cell-free superoxide-generating system was also used to demonstrate that GNP and its derivatives did not promote superoxide scavenging or scavenge superoxide directly (Figure J in S1 File).

The mitochondrial membrane potential is regulated by UCP2 in intact cells. The mitochondrial membrane potential is increased due to a lack of UCP2 or the removal of endogenous superoxides in dispersed islet cells and in many types of cancer cells. A mitochondrial membrane potential-sensitive fluorescent dye (JC-1) was used to investigate whether the GNP derivatives had the same ability as GNP to induce changes in the mitochondrial membrane potential. GNP, 10-GNP1 and 10-GNP2 increased in the fluorescence intensity of mitochondrial JC-1 over time, and 10-GNP1 and 10-GNP2 also significantly increased fluorescence intensity of JC-1 compared with the control group. However, neither 1-GNP1 nor 1-GNP2 affected this fluorescence intensity (Fig 5E). These findings demonstrate that the inhibitory effects on UCP2 were related to an increase in the mitochondrial membrane potential. Thus, the 1-OH of GNP was deduced to be the key site of its biological effects.

## Discussion

In recent years, GNP has been reported to have potential anti-cancer effects. Many studies have revealed that various tumor cell lines undergo apoptosis following GNP treatment. However, the relationship between its structure and its therapeutic effects has not yet been elucidated. Apoptosis is associated with several characteristic morphological changes, including the appearance of more rounded, shrunken, and suspended cells, and leads to an eventual reduction in the number of surviving cells. The typical characteristics of apoptosis were observed after cells were treated with GNP and 10-OH-substituted derivatives, whereas cellular morphology did not change after cells were treated with 1-OH-substituted derivatives. The IC_50_ values of GNP and the 10-OH-substituted derivatives ranged from 219 μM to 290.5 μM, but the values of the 1-OH-substituted derivatives exceeded 400 μM. Treatment with GNP and the 10-OH-substituted derivatives significantly altered the cell cycle profile and increased the number of apoptotic cells, demonstrating that the decrease in cell viability was caused by the induction of apoptosis and was associated with cell cycle arrest. These results are consistent with those of a previous report that compared the effects of GNP and 1-GNP1 in hepatocellular carcinoma cells [12]. HepG2, a hepatocellular carcinoma cell line, was chosen to compare the inhibitory effects of GNP and its derivatives, and the results were consistent with those obtained for the Panc-1 cells, indicating that the inhibitory effect was not cell line dependent. Thus, we speculate that the active group of GNP is 1-OH and not 10-OH.

A reduction in the number of surviving cells may be caused by the induction of cell death and/or the inhibition of cell proliferation. GNP and 10-OH-substituted derivatives could induce Panc-1 apoptosis, as demonstrated by annexin-V/PI staining. Microscopy revealed that the number of rounded, shrunken, and suspended cells were increased whereas the number of whole cells was significantly reduced. Based on Hoechst 33258 staining, treatment with GNP and its 10-OH-substituted derivatives also appeared to increase nuclear size, an effect that was caused by G_2_/M arrest. GNP and 10-GNP2 caused G_2_/M arrest in Panc-1 cells, whereas 1-GNP1 and 1-GNP2 had no effects on the cell cycle of Panc-1 cells. The size of the nucleus in cells in the G_2_/M phase is reportedly larger than that in cells in the G_0_/G_1_ phase, which may be because cellular components, including DNA and proteins, are naturally enlarged in preparation for mitosis. A similar phenomenon has been observed for the etoposide- and temozolomide-induced arrest of HCT116 cells and glioma cells in G_2_/M phase [36, 37].

The effect of 10-GNP1 on G_2_/M arrest was not obvious when cell cycle progression was analyzed, but 10-GNP1 could induce a higher apoptosis rate than GNP and 10-GNP2 at 72 h. This result could be due to the different chemical structures of 10-GNP1, 10-GNP2 and GNP. As 10-OH was replaced with trimethylacetyl, which is a large group, the solubility of 10-GNP1 in the cell culture medium was much lower than that of 10-GNP2 and GNP in cell culture medium. In addition, the different chemical structures would also cause dissimilarity in the absorption rates of 10-GNP1, 10-GNP2 and GNP in Panc-1 cells. At 24 h, the G_2_/M- arrested population of the 10-GNP1-treated cells was not as large as those of 10-GNP2- and GNP-treated cells, but we found that the size of this cell population increased to 34.06% at 48 h, demonstrating that different amounts of time were required for 10-GNP1, 10-GNP2 and GNP to reach their full effect in Panc-1 cells. The IC_50_ of 10-GNP1 was lower than those of 10-GNP2 and GNP at 72 h. The inconsistent results may be due to the low solubility and slower absorption rate of 10-GNP1, and similar effects were also observed with another treatment at 24 h for the detection of mitochondrial membrane potential.

Pancreatic cancer is considered one of the most lethal cancers, and its mortality rate is nearly equal to its incidence rate. Mitochondrial UCP2 has been reported to be over-expressed in pancreatic cancer tissues compared with adjacent normal tissues [38]. UCP2 over-expression is a possible strategy adopted by cancer cells for protection from excess ROS production. UCP2 inhibition could represent a therapeutic strategy, especially for the treatment of chemo-resistant tumors. The disruption of UCP2 transcription by a specific siRNA and the inhibition of UCP2 function by specific compounds, such as GNP, could also be useful clinical strategies. The inhibition of UCP2 allows ROS accumulation and genomic instability, while the negative regulation of mitochondrial ROS production through UCP2 over-expression usually occurs during cancer development, chemo-resistance, and tumor metastasis to defend cancer cells from apoptosis. The antioxidant effect of UCP2 plays a vital role in the resistance of pancreatic cancer cells to standard chemotherapy. Moreover, the mitochondrial membrane potential is increased due to a lack of UCP2 or the removal of endogenous superoxides in dispersed islet cells and in many types of cancer cells. Our results showed that the apoptosis of Panc-1 cells induced by GNP, 10-GNP1, and 10-GNP2 was related to the inhibition of UCP2 function, which in turn led to an increase in ROS production and the mitochondrial membrane potential, whereas the GNP derivatives with 1-OH substitutions (1-GNP1 and 1-GNP2) lost all of these abilities. Thus, the 1-OH in GNP was deduced to be the key site for its biological effects.

## Conclusions

This study is the first to identify the key active site in the structure of GNP. The results indicated that the 1-OH of GNP is very important for its cytotoxic effect on Panc-1 cells. The derivatives 10-GNP1 and 10-GNP2, which retained the 1-OH group, exhibited inhibitory effects similar to those of GNP. In contrast, the derivatives 1-GNP1 and 1-GNP2, in which 1-OH was substituted with other groups, did not inhibit Panc-1 cell growth. The HepG2 cell line was used to show that the inhibitory effects were not cell line dependent. Additionally, the inhibitory effects were studied by performing a cell proliferation assay, fluorescence microscopy, and flow cytometric analyses of the cell cycle and cell apoptosis. The mechanism of apoptosis was related to the inhibition of UCP2 function, which in turn led to increases in ROS production and the mitochondrial membrane potential. Based on these conclusions, new GNP derivatives can be developed in which the 1-OH group and their biological activities are maintained. The results of this study support the use of UCP2 inhibitors in anti-tumor strategies for the treatment of pancreatic adenocarcinoma and provide novel insight into the structure of GNP.

## Supporting Information

S1 File Plasmid transfection rate determined by cytofluorimetric analysis (Figure A). UCP2 over-expression or silencing, as measured by Western blotting (Figure B). UCP2 gene silencing, as measured by qPCR (Figure C). ^1^HNMR spectrum of 1-GNP2 (Figure D). ^13^CNMR spectrum of 1-GNP2 (Figure E). ^1^HNMR spectrum of 10-GNP1 (Figure F). ^13^CNMR spectrum of 10-GNP1 (Figure G). ^1^HNMR spectrum of 10-GNP2 (Figure H). ^13^CNMR spectrum of 10-GNP2 (Figure I). The effects of GNP and its derivatives on direct superoxide scavenging and the promotion of superoxide scavenging (Figure J).
(DOC)
