# Supplementary material for: The Hydroxyl at Position C1 of Genipin Is the Active Inhibitory Group that Affects Mitochondrial Uncoupling Protein 2 in Panc-1 Cells
Source: PLoS One. 2016 Jan 15;11(1):e0147026. doi: 10.1371/journal.pone.0147026 (PMC4714807; doi:10.1371/journal.pone.0147026)
Supplement: S1 File — Plasmid transfection rate determined by cytofluorimetric analysis (Figure A). UCP2 over-expression or silencing, as measured by Western blotting (Figure B). UCP2 gene silencing, as measured by qPCR (Figure C). 1HNMR spectrum of 1-GNP2 (Figure D). 13CNMR spectrum of 1-GNP2 (Figure E). 1HNMR spectrum of 10-GNP1 (Figure F). 13CNMR spectrum of 10-GNP1 (Figure G). 1HNMR spectrum of 10-GNP2 (Figure H). 13CNMR spectrum of 10-GNP2 (Figure I). The effects of GNP and its derivatives on direct superoxide scavenging and the promotion of superoxide scavenging (Figure J). (DOC) [file pone.0147026.s001.doc]

**S1 File.**


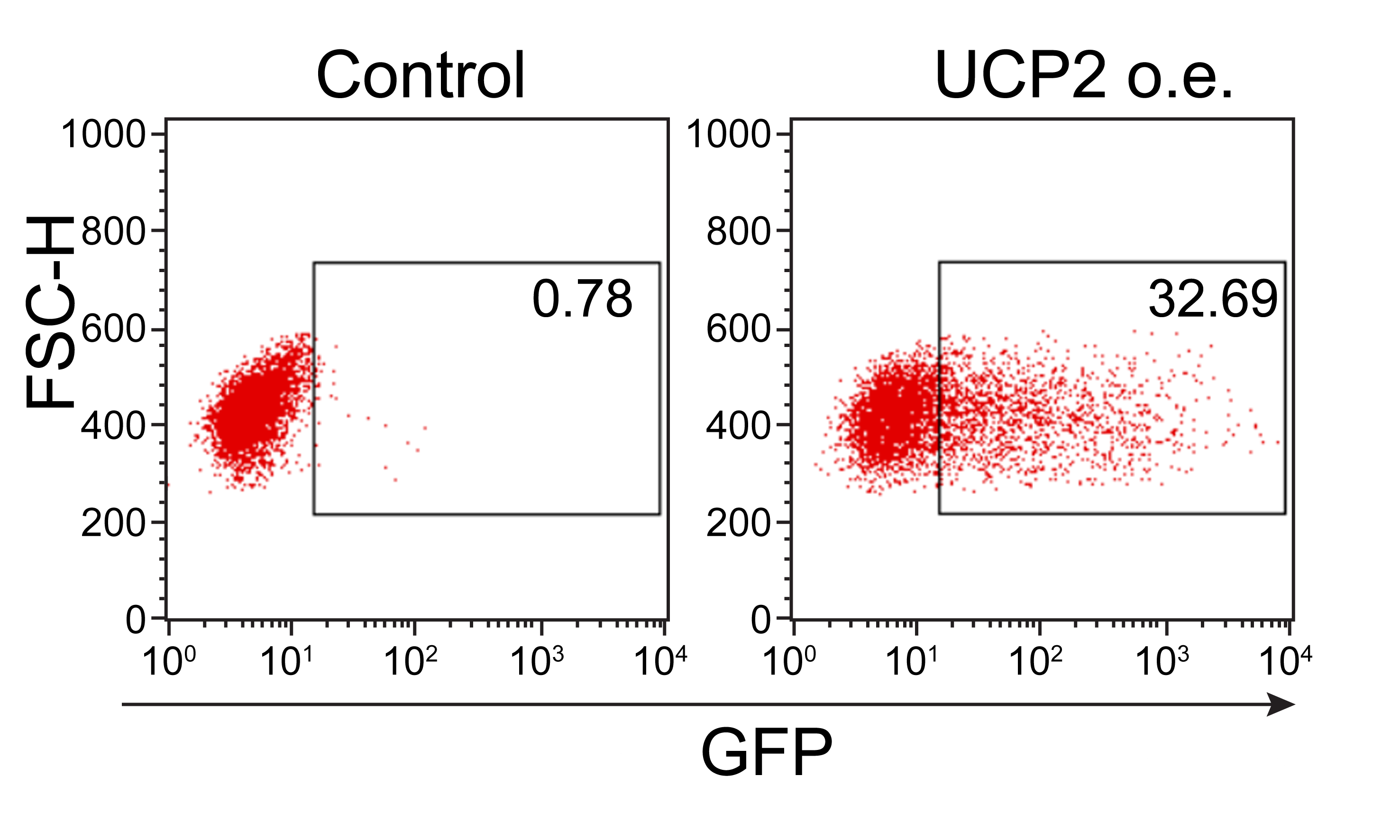


**Figure A Plasmid transfection rate determined by cytofluorimetric analysis.**


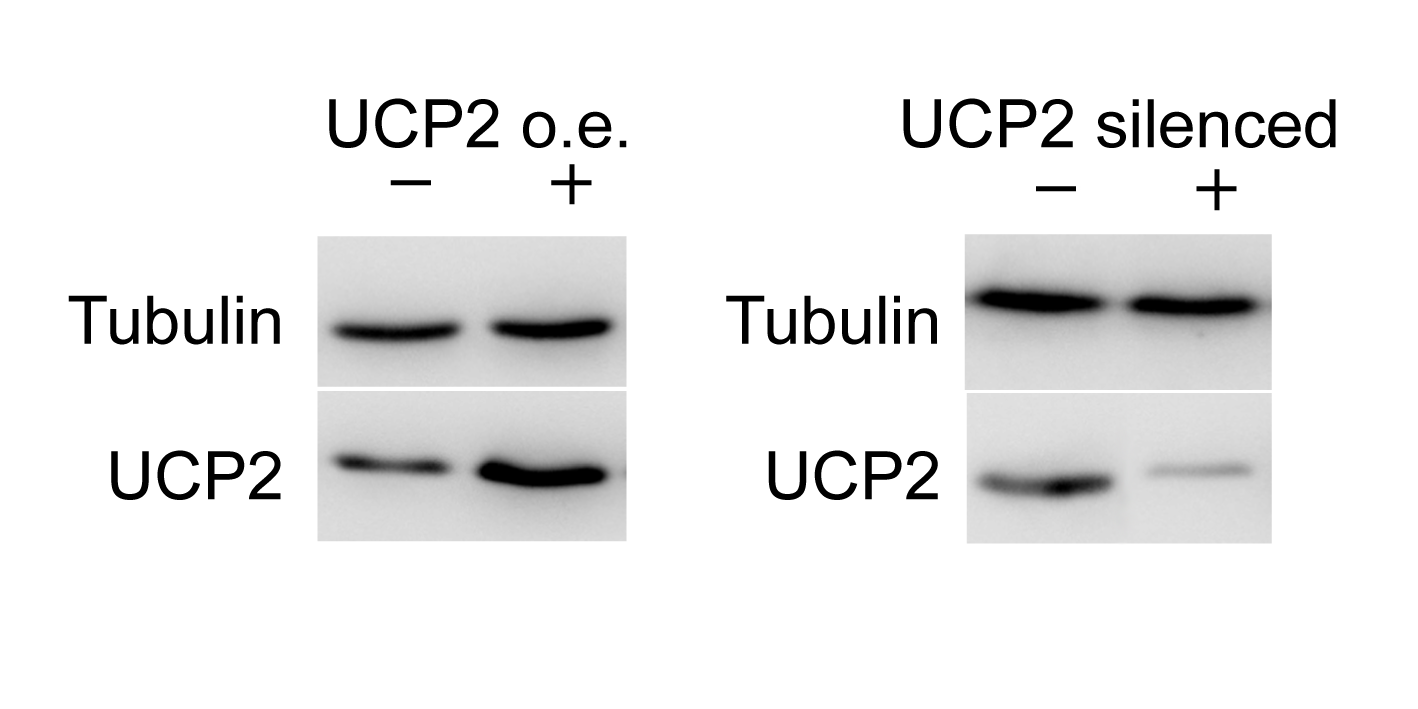


**Figure B UCP2 over-expression or silencing, as measured by Western blotting.**

After UCP2 was over-expressed and silenced in Panc-1 cells, the cells were lysed with RIPA buffer to measure UCP2 expression by Western blot analysis.


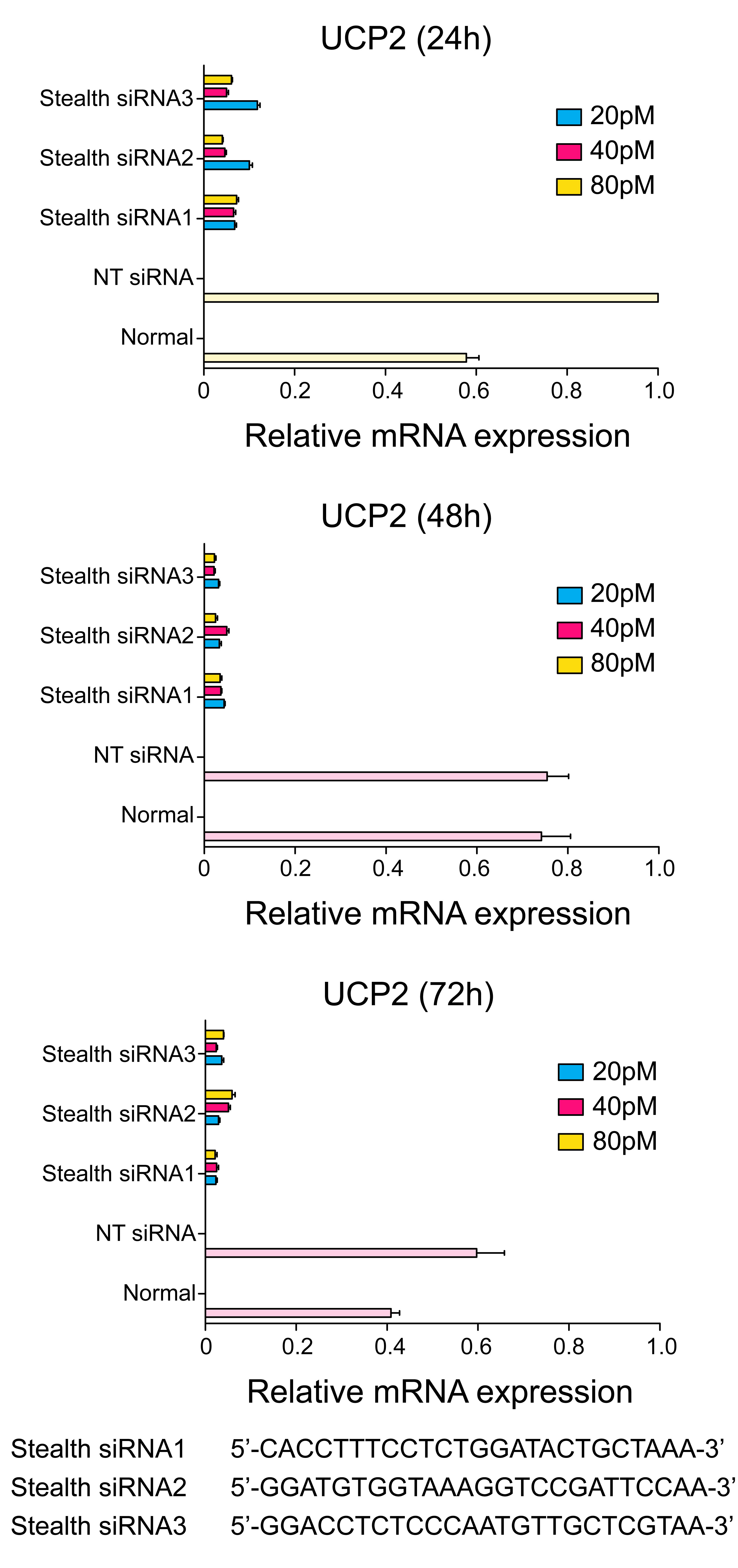


**Figure C UCP2 gene silencing, as measured by qPCR.**

After the UCP2 gene was silenced by stealth siRNA, the level of UCP2 transcription was determined by quantitative RT-PCR analysis. The data are the mean (±SE) of three independent experiments performed in triplicate.
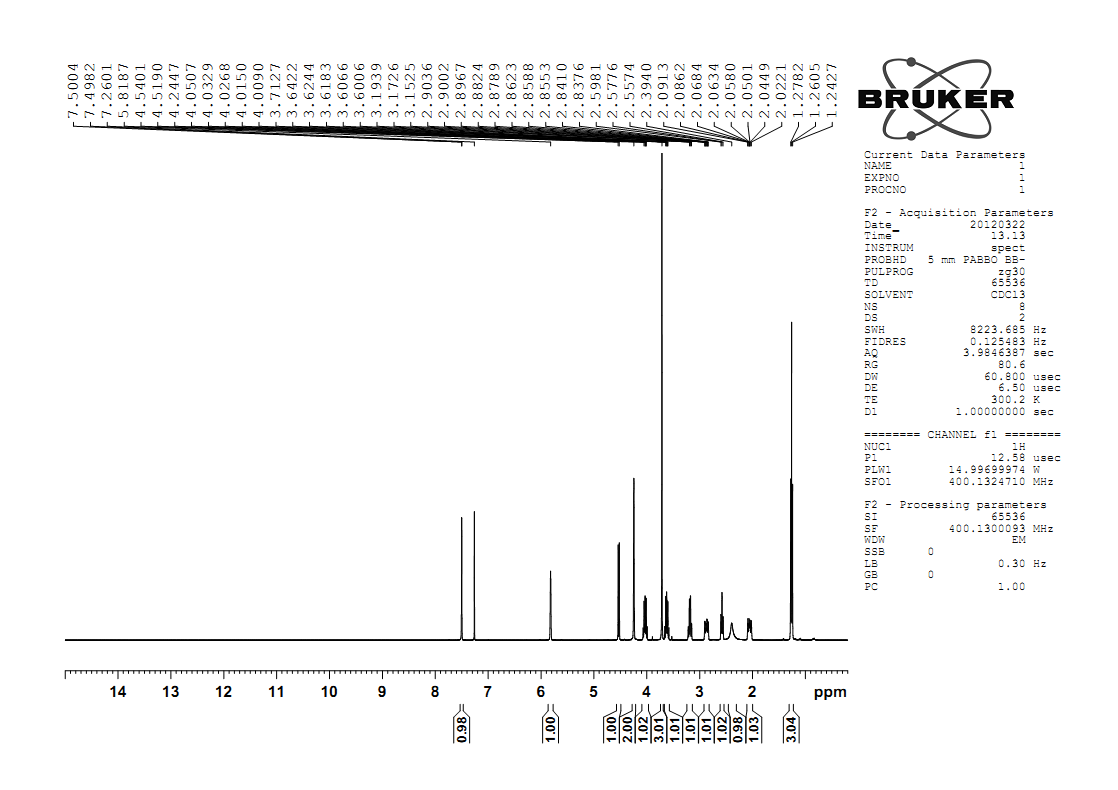


**Figure D ^1^H-NMR spectrum of 1-GNP2**


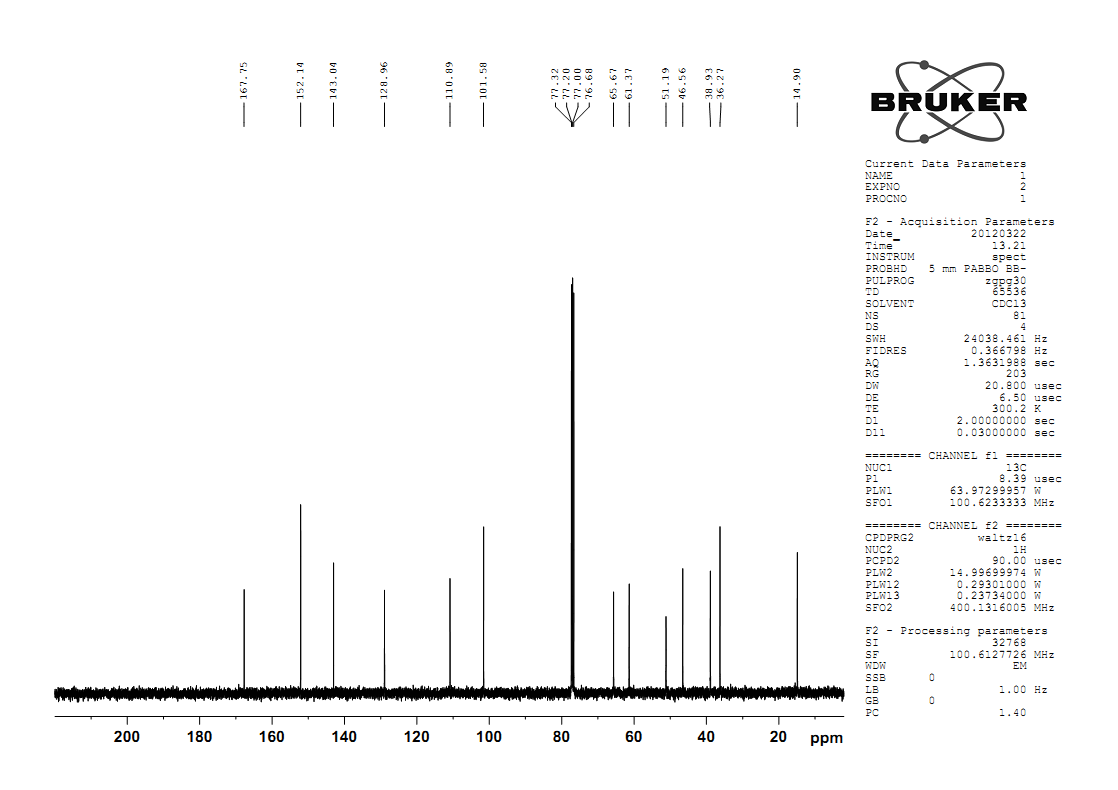


**Figure E ^13^C-NMR spectrum of 1-GNP2**


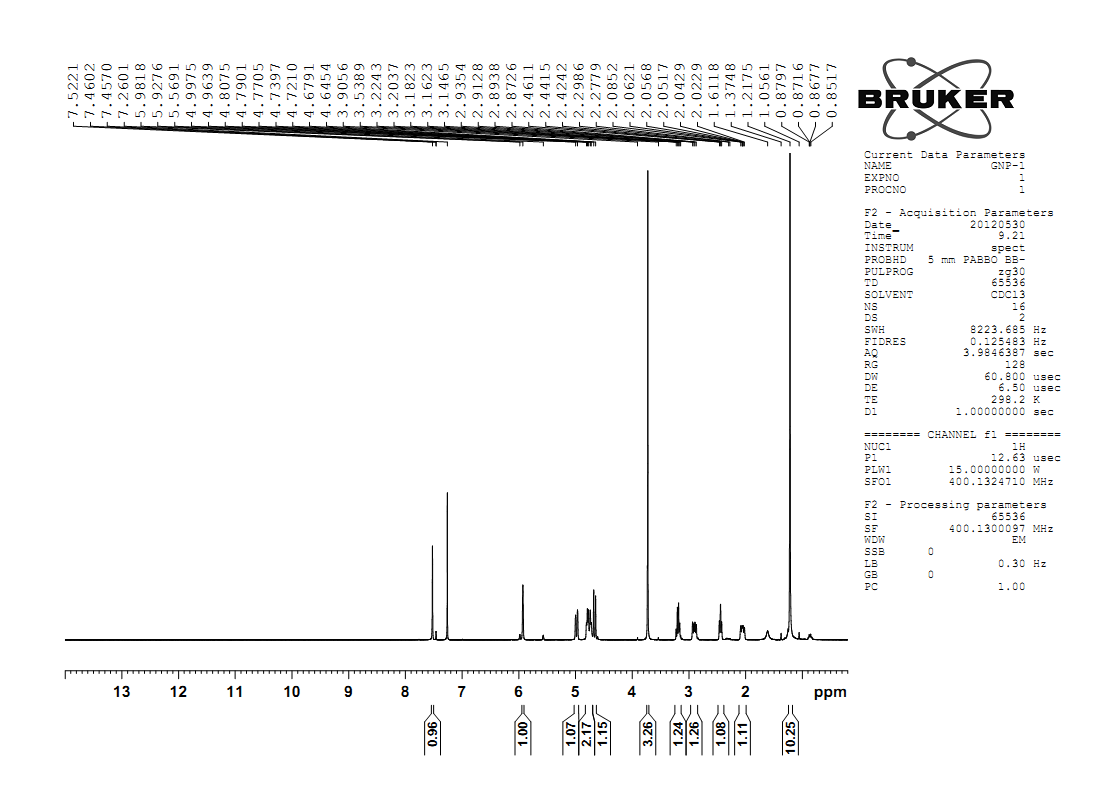


**Figure F ^1^H-NMR spectrum of 10-GNP1**


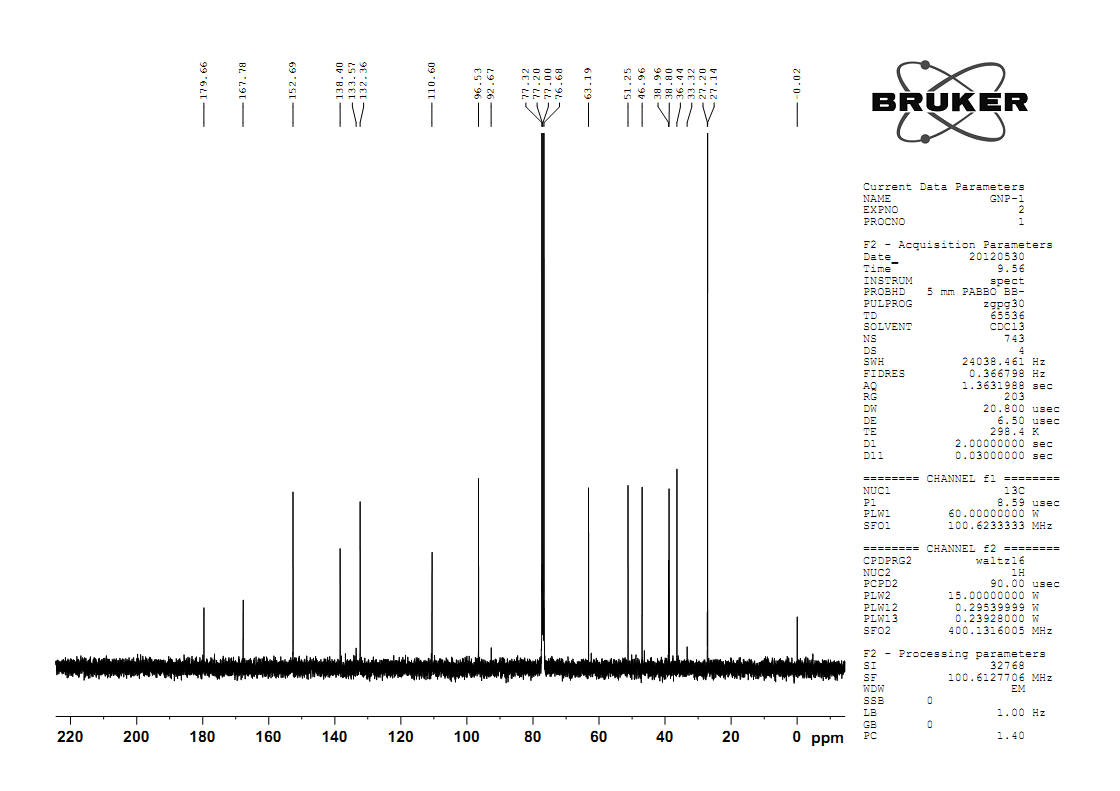


**Figure G ^13^C-NMR spectrum of 10-GNP1**

**
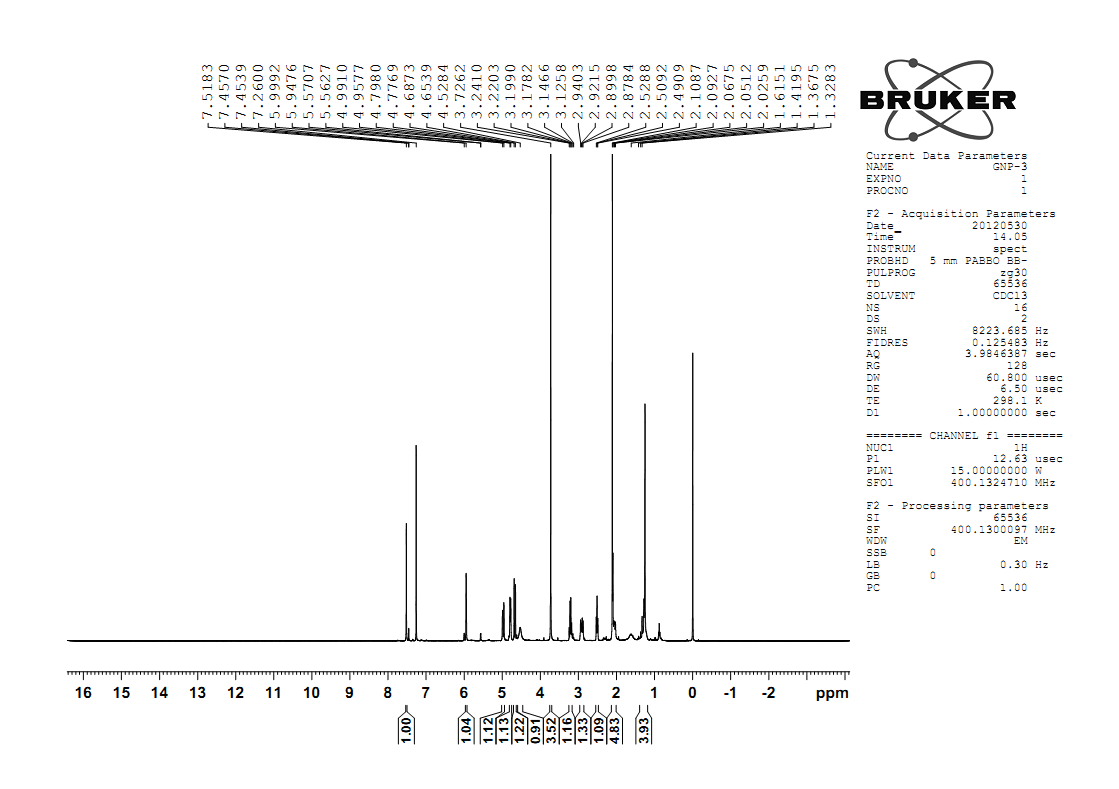
**

**Figure H ^1^H-NMR spectrum of 10-GNP2 acid-genipin
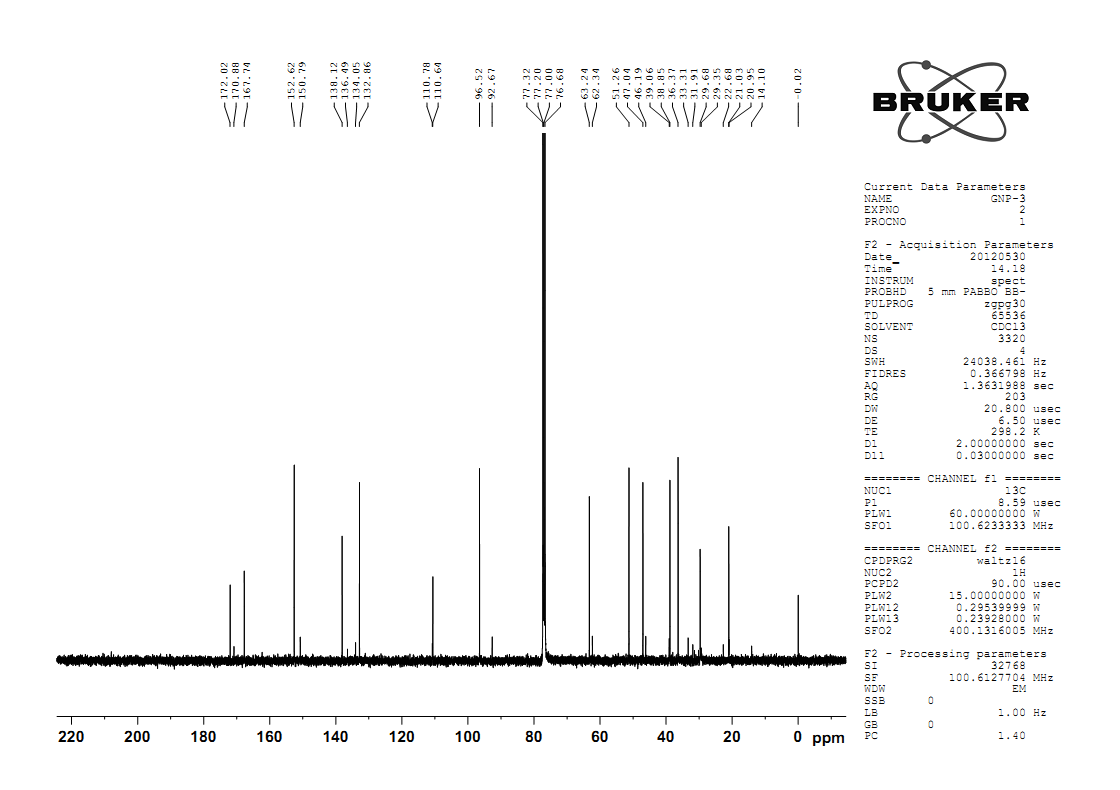
**

**Figure I ^13^C-NMR spectrum of 10-GNP1**

**^1^H-NMR and ^13^C-NMR data for 1-GNP2, 10-GNP1, and 10-GNP2**

**1-GNP2:** ^1^H-NMR (CDCl_3_): δ7.50 (s, 1H, 3-H), 5.82 (s, 1H, 7-H), 4.53 (d, 1H, j=8.4, 1-H), 4.24 (s, 2H, 10-H), 4.07-4.01 (m, 1H, 1-Ha), 3.72 (s, 3H, O-CH_3_), 3.66-3.60 (m, 1H, 1-Hb), 3.21-3.15 (q, 1H, j=7.9, 5-H), 2.90-2.83 (m, 1H, 6-Ha), 2.59-2.55 (t, 1H,, 9-H), 2.39 (br, 1H, -OH), 2.09-2.01 (m, 1H, 6-Hb), and 1.28-1.24 (t, 3H, J=7.1, 2’-H); ^13^C-NMR (CDCl_3_): δ167.7 (11-C), 152.1 (3-C), 143.0 (8-C), 129.0 (7-C), 110.9 (4-C), 101.6 (1-C), 65.7 (1’-C), 61.4 (10-C), 51.2 (-OCH_3_), 46.6 (9-C), 38.9 (6-C), 36.3 (5-C), and 14.9 (2’-C).

**10-GNP1:** ^1^H-NMR (400 MHZ CDCl_3_): δ7.52 (s, 1H, 3-H), 5.92 (s, 1H, 7-H), 4.98 (d, 1H, J=13.4, 10-Ha), 4.80-4.77 (m, 1H, 1-H), 4.66 (d, J=13.4, 1H, 10-Hb), 3.72 (s, 3H, -OCH3), 3.22-3.14 (m, 1H, 5-H), 2.93-2.87 (m, 1H, 6-Ha), 2.44 (t, J=7.8 HZ, 9-H), 2.08-2.03 (m, 1H, 6-Hb), and 1.21 (s, 9H-PIV); ^13^C-NMR (100 MHZ, CDCl_3_): δ179.6 (piv-c), 167.8 (11-c), 152.7 (3-c), 138.4 (8-c), 132.8 (7-c), 110.6 (4-c), 96.5 (1-c), 63.2 (10-c), 51.3 (-OCH_3_), 47.0 (9-c), 38.9 (6-c), 36.4 (5-C), and 27.1 (PIV-CH_3_). **10-GNP2:** ^1^H-NMR (400 MHZ CDCl_3_): δ7.52 (s, 1H, 3-H), 6.00 (s, 1H, 7-H), 4.97 (d, 1H, j=13.3, 10-Ha), 4.78 (d, 1H, j=8.4, 1-H), 4.67 (d, 1H, j=13.2, 10-Hb), 3.72 (S, 3H, 1’’-CH_3_), 3.24-3.12 (m, 1H, 5-H), 2.94-2.87 (m, 1H, 6-Ha), 2.51 (t, J=7.8 HZ, 1H, 9-H), 2.10 (s, 3H, 2’-CH_3_), and 2.10-2.02 (m, 1H, 6-Hb); ^13^C-NMR (100 MHZ, CDCl_3_): 172.0 (1’-C), 167.7 (11-C), 152.6 (3-C), 138.1 (8-C), 132.8 (7-C), 110.6 (4-C), 96.5 (1-C), 63.2 (10-C), 51.2 (1’’-C), 47.0 (9-C), 38.8 (6-C), 36.4 (5-C), and 22.6 (2’-C).


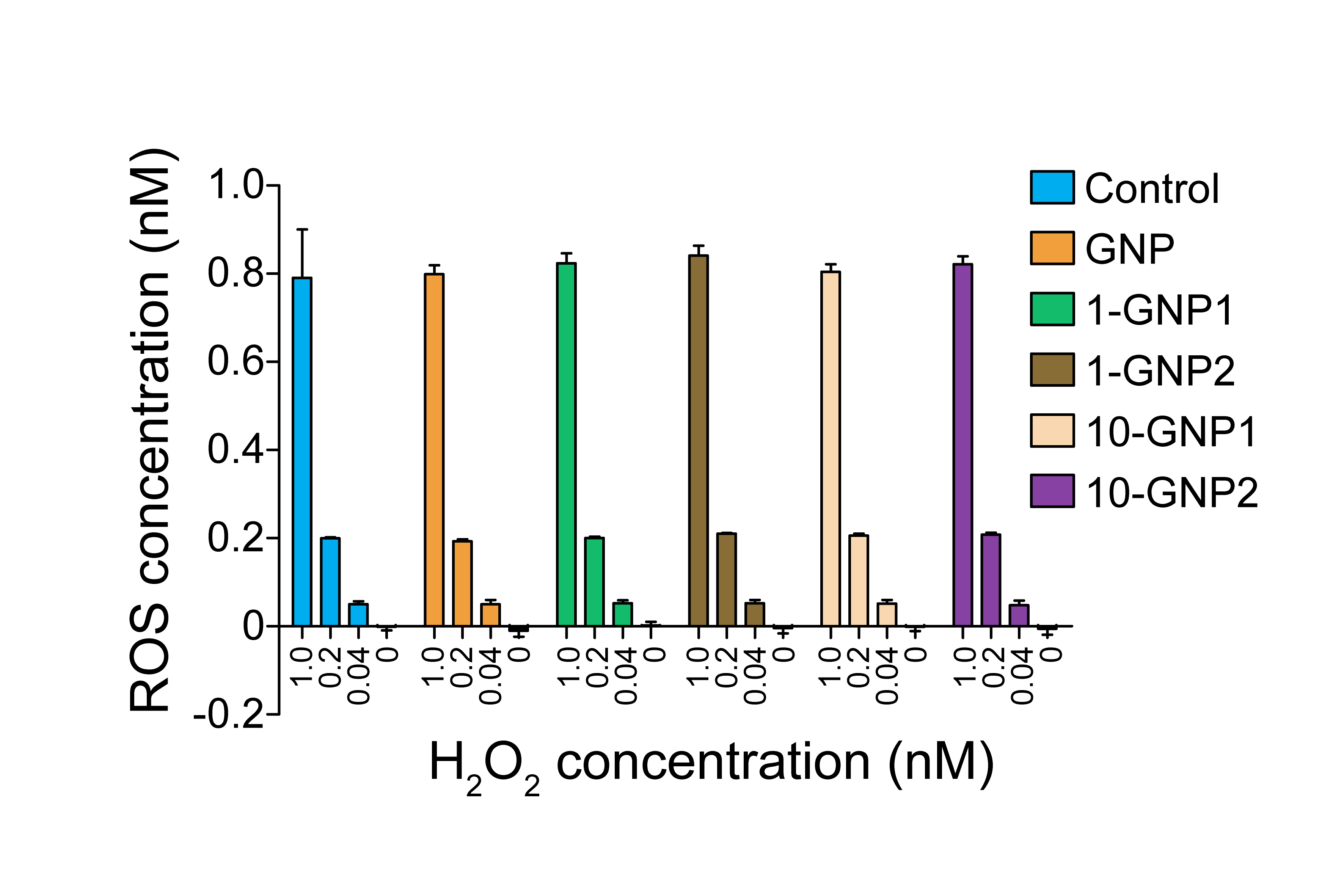


**Figure J The effects of GNP and its derivatives on direct superoxide scavenging and the promotion of superoxide scavenging.**

GNP and its derivatives (at 200 μM) were pre-treated with H_2_O_2_ (1, 0.2, and 0.04 nM) in an *in vitro* cell-free system for 1 h. ROS concentrations were measured using a ROS assay kit. Statistical analysis: GNP and its derivatives *vs.* control (pre-treatment with the same concentration of H_2_O_2_). For each concentration of H_2_O_2_ (1, 0.2, and 0.04 nM), there were no significant differences among the six groups. The values are the mean (±SE) of three independent experiments performed in triplicate.
